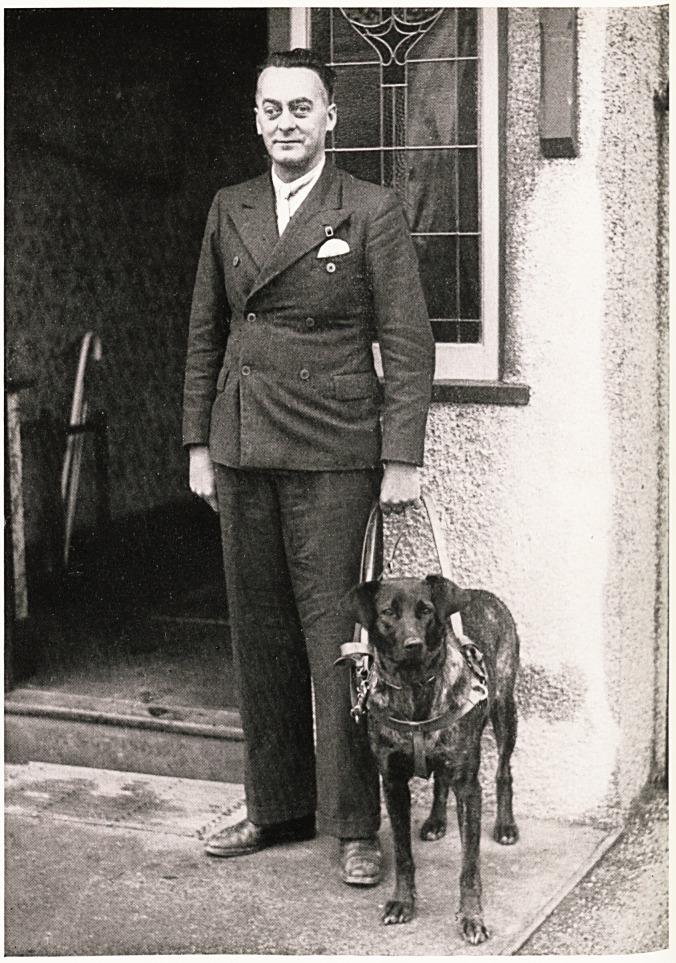# A Guide Dog for the Blind

**Published:** 1938

**Authors:** F. Wilfred Willway

**Affiliations:** Assistant Surgeon West End Hospital for Nervous Diseases; Surgical Registrar Bristol Royal Infirmary


### A GUIDE DOG FOR THE BLIND.

BY

F. Wilfred Willway, F.R.C.S.,

*Assistant Surgeon West End Hospital for Nervous Diseases; Surgical Registrar Bristol Royal Infirmary.*

---

It is thought that readers might be interested in this short account of the first guide dog for the blind in the City and County of Bristol or in Gloucestershire.

In 1936, "R.H.," a man of 42, married, with a family, was taken ill with symptoms of raised intracranial pressure. He was a draughtsman at Filton Aeroplane Works, in a good position.

Investigations at the Royal Infirmary (Dr. Richard Clarke, Professor Rendle Short and the writer) all pointed to the presence of a "space-occupying lesion." Ventriculography revealed severe internal hydrocephalus. His vision was nil in the right eye, and the field was markedly restricted in the left eye.

On 6th November, 1936, exploratory craniotomy was performed and finally a right-sided sub-temporal decompression. The headache was at once relieved, the vomiting gradually disappeared, but vision rapidly failed, the patient being left with bilateral secondary optic atrophy. For the past two years he has remained "cured but blind" in the lay sense of the term even if one has to be more guarded in its scientific application. It is not necessary to enlarge on the horrors of his position, suddenly deprived of work, amusement, income, with a family to educate. The

"horror of darkness" which every blinded man undergoes was particularly troublesome here in that neither he nor his wife believed he had more than a few months to live.

With the passage of time and its healing influence, "R.H." began to believe that he was indeed "cured," and then the writer decided to get a guide dog for him, believing that a doctor should treat the environment when he cannot treat the patient directly.

There is in this country a charity termed "The Guide Dogs for the Blind Association," with its training centre at Wallasey, Cheshire. The writer applied for a dog for his patient, and (thanks to help from many friends) the man was accepted and finally sent for. At Wallasey bitches (mainly Alsatians) are trained for approximately three months. The bitch must be absolutely trustworthy as well as intelligent, as the blind man places absolute reliance on the dog's guidance and will unhesitatingly follow the dog's lead. The patient had to spend a period at Wallasey, where he was taught the system, trained with his dog and finally passed his tests and was sent home to begin his adventures. The dog wears a semi-rigid harness with a handle which the patient can comfortably grasp (see photograph). The dog also wears a chain or lead. When the dog is on the lead she knows she is "off duty"—the stand-easy position. When the harness is held the dog is "on duty," ready to guide and care for her master. The dog understands the commands "Left," "Right," and "Forward," but with this significant addition—she will not obey such commands until it is safe to do so. The writer was greatly impressed to see "R.H." with "Nigger" at the curb of a fairly busy road—six times he commanded "Forward, Nigger!" and five times the dog did not move, because of cars or cycles. On the sixth occasion she moved off in good order, not checking until she reached the opposite curb, which the patient then felt for with his foot. Clearly, intelligent obedience rather than simple obedience is required. "R.H." told the writer that it might be five minutes before the cautious dog would take him across a very busy road like the Gloucester Road. Obviously, a blind man cannot dash across in and out of vehicles.

"R.H." has had his dog some two and a half months and is only just at the beginning of his capabilities, but already he has the following achievements :

(1) He goes out twice a day, wet or fine, a distance of about two miles. This is in his regulations, as these big dogs love exercise.

(2) He crosses the Gloucester Road by himself and has no anxiety in such a hazardous procedure. As mentioned above, he may first have to pause for a long time.

(3) He has been to the Royal Infirmary and back from Filton alone with his dog.

(4) He daily does the shopping with his dog in a district with which he is, of course, familiar.

It is proper, at this stage, to add some of the things the animal will *not* do. Thus, "R.H." does not whisper "Fishmonger" in the animal's ear, whereupon the sagacious hound leads his master faithfully to the nearest fish-and-chip shop! In fact, he does not even say "Home, Nigger!" when he turns back on one of his strolls. It is for "R.H." to take the dog, not the dog take him. The dog's duty is solely to safeguard the owner. Of course, dogs soon get used to habit, and if, for example, "R.H." every Sunday afternoon went to hear the open-air speakers on

Durdham Down, it is quite probable that if he said, after some weeks, "Downs, Nigger!" then the intelligent animal would set off for the meetings! This is not unreasonable, as every dog-owner knows how a dog appreciates habit and routine. Again, the dog does not know the way, and the patient has at times to ask where he is, and when told knows then what directions to give to his dog. The writer has been asked why bitches are selected. It is because they are more docile and affectionate than dogs and, also, excepting when on "heat," they do not show an interest in other dogs. As a bitch only "comes on heat" twice in the year for three weeks at a stretch, this is not a serious drawback, though at these times they are difficult. Ordinarily, the bitch ignores the advances of dogs.

In conclusion, it may be said that the improvement the Guide dog has brought to "R.H.," both psychologically and in general health, is difficult to describe in a short space in a public journal. Surely, such work is a part of the healing art?

The writer will be pleased to give any further information he can to interested readers. By the editor's courtesy, he is allowed to say that he will gladly forward to the Guide Dogs for the Blind Association, any donations that charitable readers may feel disposed to give to such a laudable work. The training of a dog and man works out at about £60.

## Figures and Tables

**Figure f1:**